# Reopenable-clip over-the-line method used inside a submucosal pocket during rectal endoscopic submucosal dissection for a full-thickness defect

**DOI:** 10.1055/a-2228-7345

**Published:** 2024-01-23

**Authors:** Tatsuma Nomura, Shinya Sugimoto, Ryutaro Matsushima, Taishi Temma, Jun Oyamada, Keiichi Ito, Akira Kamei

**Affiliations:** 1 37071 Department of Gastroenterology, Ise Red Cross Hospital, Ise, Japan; 2 38367 Gastroenterology, Mie Prefectural Shima Hospital, Shima, Japan

Colorectal endoscopic submucosal dissection (ESD) is a feasible method for en bloc resection of large tumors; however, en bloc resection of Paris type 0-Is lesions may require muscle layer dissection resulting in intraoperative perforation 1 . We previously reported a “reopenable-clip over-the-line method inside a submucosal pocket” (ROLM-SP) that allows closure of large full-thickness defects in the third space 2 . Here, we describe the first use of ROLM-SP for early rectal cancer ( Video 1 ).

Full-thickness closure using the reopenable-clip over-the-line method inside a submucosal pocket to close a defect after endoscopic submucosal dissection for a rectal Paris type 0-Is tumor.Video 1

The patient was an 86-year-old woman with a 40-mm Paris type 0-Is tumor in the upper rectum ( Fig. 1 ). We performed en bloc resection using the pocket-creation method with the calibrated, small-caliber tip, transparent (CAST) hood and saline immersion 3 4 . First, a submucosal pocket was created through a mucosal incision. Muscle layer perforation occurred when the central muscle layer was dissected to achieve a negative vertical margin. A 12-mm full-thickness defect was created in the pocket, with there being no fluid leakage into the abdominal cavity because of the tissue outside the muscle layer.

**Fig. 1:**
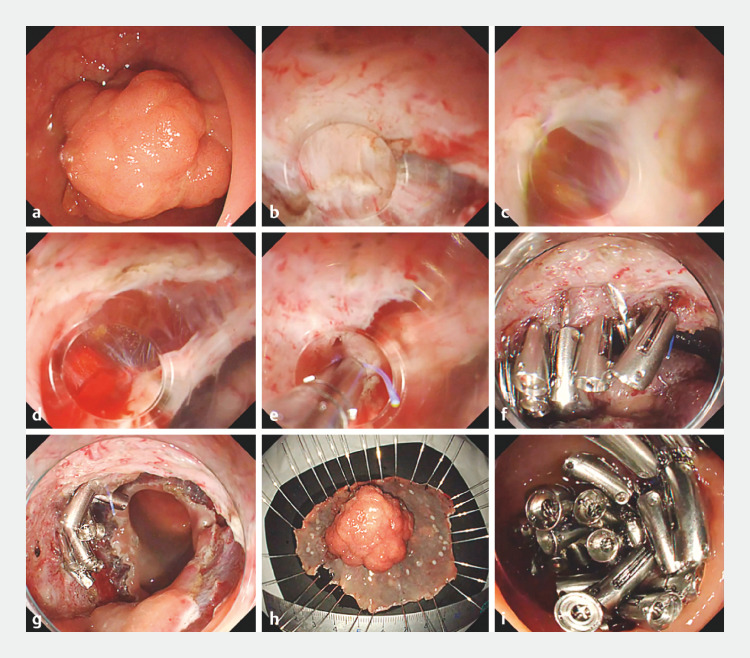
Endoscopic images of full-thickness closure using the reopenable-clip over-the-line method in a submucosal pocket (ROLM-SP) in the rectum showing: **a** an early rectal cancer in the upper rectum at 40 mm; **b** dissection of the muscle layer beneath the center of the tumor using the pocket-creation method; **c,d** a 12-mm full-thickness defect below the center of the tumor that was created during dissection; **e,f** full-thickness defect closure performed in the mucosal pocket using ROLM; **g** appearance after residual mucosa had been excised and the tumor completely resected; **h** macroscopic appearance of the excised tumor, which was revealed to be an intramucosal carcinoma; **i** complete closure of the mucosal full-thickness defect.

The full-thickness defect was closed using the ROLM-SP. First, a clip with line was inserted through the accessory channel. The clip was placed on the muscle layer at the edge of the full-thickness defect. A line from the accessory channel was threaded through the hole in one of the teeth of the clip 5 . The clip was then placed on the muscle of the contralateral edge of the full-thickness defect. Repeat placement of a clip with a line through the tooth hole onto the muscle at the edge of the defect resulted in progressive closure of the defect. The full-thickness defect was completely closed within the submucosal pocket. The remaining mucosa was excised and the tumor was removed. The remaining mucosal defect was completely closed using the ROLM.

The patient was discharged without there being any adverse events. ROLM-SP is an effective technique for closing large full-thickness defects in the third space.

Endoscopy_UCTN_Code_TTT_1AQ_2AG

## Notes

Endoscopy E-Videos https://eref.thieme.de/e-videos
*E-Videos* is an open access online section of the journal *Endoscopy* , reporting on interesting cases and new techniques in gastroenterological endoscopy. All papers include a high-quality video and are published with a Creative Commons CC-BY license. Endoscopy E-Videos qualify for HINARI discounts and waivers and eligibility is automatically checked during the submission process. We grant 100% waivers to articles whose corresponding authors are based in Group A countries and 50% waivers to those who are based in Group B countries as classified by Research4Life (see: https://www.research4life.org/access/eligibility/ ). This section has its own submission website at https://mc.manuscriptcentral.com/e-videos .
